# Acceptability and preliminary effectiveness of a single-arm 12-week digital behavioral health intervention in patients with knee osteoarthritis

**DOI:** 10.1186/s12891-023-06238-8

**Published:** 2023-02-17

**Authors:** Kristine Godziuk, Carla M. Prado, Maira Quintanilha, Mary Forhan

**Affiliations:** 1 Department of Agricultural, Food and Nutritional Science, Faculty of Agricultural, Life and Environmental Sciences, University of Alberta, 2-004 Li Ka Shing Centre, Edmonton, AB T6G 2P5 Canada; 2 Department of Occupational Science and Occupational Therapy, University of Toronto, Toronto, ON Canada

**Keywords:** Osteoarthritis, Arthroplasty, e-health, Multimethods, Nutrition, Exercise, Mindfulness, Knee osteoarthritis, Digital intervention, Behavioral intervention

## Abstract

**Background:**

Digital health interventions may improve osteoarthritis (OA) management. This study evaluated the acceptability and preliminary effectiveness of a multimodal digital nutrition, exercise, and mindfulness intervention in adults with knee OA.

**Methods:**

Adults with advanced knee OA and an orthopaedic referral were invited to self-enroll in a pragmatic 12-week single-arm intervention. OA-focused nutrition and exercise resources were delivered weekly by email, and secondary components accessed on-demand (web-platform, webinars, and nutrition consultation). Acceptability was assessed by qualitative interview data and completion rates. Preliminary effectiveness on clinical outcomes was assessed by change in health-related quality of life, well-being, mindfulness, self-efficacy, and interest in total knee arthroplasty (TKA) between baseline and 12-weeks.

**Results:**

*N* = 102 patients self-enrolled (73.5% female, age 64 ± 7 years, body mass index 32.9 ± 7.3 kg/m^2^); *n* = 53 completed the 12-week intervention (71.7% female, age 65 ± 7 years, body mass index 33.4 ± 6.3 kg/m^2^). Acceptability was demonstrated by positive perceptions of tailored intervention resources. In study completers, health-related quality of life components of pain and physical functioning domains improved at 12-weeks [change in SF36 4.4 (95%CI 0.2–8.6), *p* = 0.016, and 6.7 (95%CI 2.7–10.7), *p* < 0.001, respectively]. Self-efficacy for managing daily activities improved [change in PROMIS T-score 4.4 (95%CI 2.8–6.0), *p* < 0.001].

**Conclusion:**

A 12-week digital multimodal intervention for knee OA was acceptable to patients and shows preliminary effectiveness in improving self-efficacy, aspects of quality of life, and decreasing interest in TKA. Digital behavioral interventions for knee OA may be an acceptable approach to improve patient outcomes and OA self-management while potentially reducing utilization of costly health system resources.

**Supplementary Information:**

The online version contains supplementary material available at 10.1186/s12891-023-06238-8.

## Significance

- A digital multimodal intervention encompassing nutrition, exercise, and mindfulness provided to patients waiting on an orthopaedic consult was acceptable to patients with preliminary evidence on improvements in quality of life components of pain and physical function.
- This low cost, simple to deliver digital health intervention may have value in decreasing uptake of costly surgical care. Further investigation is needed.
- Exercise supports delivered through the intervention were acceptable and valued by patients, while nutrition support may benefit from further customization.

## Background

Improving access to behavioral supports for nutrition, exercise, and mindfulness in patients who have osteoarthritis (OA) is an important area for advancement, as these components play a critical role to support effective chronic disease management. Nutrition and exercise have positive influences on metabolic health and body composition (i.e. fat, bone, and muscle) [1]. Exercise is essential for long-term management of OA through improving pain, strength, and physical function [2]. Adequate dietary intake can optimize body composition and prevent micronutrient deficiencies that negatively impact bone and joint health [3]. In addition, mindfulness-based stress reduction can assist patients in coping with OA-related pain [4], providing an important complement to nutrition and exercise modalities. Taken together, these non-surgical behavioral interventions can have substantive benefits in OA and contribute to avoiding or prolonging the need for surgical interventions such as joint arthroplasty [5].

There is currently access barriers within health care systems for patients to connect with health care professionals such as dietitians, exercise physiologists, physical therapists, and psychologists [6–8] to support behavior change in nutrition, exercise, and mindfulness [6–8]. While access through external private practice consultants is available, many patients are without supplementary health benefit coverage or are unable to afford out of pocket costs for this expertise. Further, the number of clinicians available in the health system within these practice areas are finite. As such, it’s unlikely they would be able to meet demand to treat all patients with OA.

Electronic or digital-health interventions have the potential to provide a solution to these access barriers [9]. Digital health interventions, as the name implies, are remotely delivered health services provided through information and communication technologies [10]. Digital interventions have been shown to be effective for pain coping skills in OA [11], and research in other clinical populations suggest that improvements in patient health outcomes after a digital intervention are comparable to results from conventional face-to-face delivery methods [12]. As such, this method allows delivery of support to patients who may not otherwise have had access to these behavioural interventions. Further, digital interventions in OA have been shown to be cost-effective compared to usual care [13].

Improving accessibility and capacity to provide services through a digital intervention could have substantive benefits at both the patient and health system levels, particularly if patients find this method of intervention delivery to be as acceptable as conventional approaches. Furthermore, this method can benefit patients waiting a considerable time before seeing an orthopaedic practitioner regarding eligibility for arthroplasty [14, 15], a problem that has grown as a result of the COVID-19 pandemic. Importantly, the pandemic also made remotely delivered care an accepted reality, providing an opportune time to explore digital interventions delivered in a pragmatic approach within orthopaedic settings. As such, this study aimed to evaluate the acceptability and preliminary effectiveness of a 12-week digital nutrition, exercise, and mindfulness self-care intervention for adults with advanced knee OA waiting for an orthopaedic consult.

## Patients and methods

### Research design

This single-arm intervention acceptability study was conducted from October 2020 to September 2021. The study design was pragmatic, including the recruitment approach, flexible intervention delivery, adherence requirements, and minimal follow-up [16]. Adults with advanced knee OA were invited to consent and self-enroll remotely and electronically. Quantitative and qualitative methods were employed in parallel in a multimethod approach. The study protocol was approved by the Research Ethics Board at the University of Alberta, Pro00102166.

### Recruitment

Invitations to participate in the study were distributed through three orthopaedic centres in Alberta, Canada, each with distinct jurisdictions for referrals. Patients who were scheduled to attend an initial knee OA consult appointment at each clinic received the invitation to participate (sample invitation included in Supplementary information A). The invitations were distributed at two clinics starting October 2020, with the third clinic joining in December 2020. As each clinic operates independently, the processes for invitation distribution differed. Clinic A provided the invitation on a separate printed page in their intake package mailed to new patients; clinic B provided the invitation through an electronic document attached to an appointment confirmation email; clinic C provided the invitation on a back page of printed clinic appointment information mailed to patients.

### Eligibility

Adults with a body mass index (BMI) ≥ 25 kg/m^2^, unilateral or bilateral knee OA and no prior joint arthroplasty were included. A link to an online BMI calculator was included on the electronic consent page for patients to self-assess their eligibility. As the study was entirely remote, height and weight were self-reported by patients and BMI was calculated. Additional inclusion criteria were ability to read English and access to home-based internet through a computer or mobile device.

### Enrollment

Patients who were interested in participating in the study could initiate self-enrollment using the provided QR code and URL on the invitation. This link directed to an electronic consent form, managed through a secure web-based Research Electronic Data Capture (REDCap) [17] system hosted at the University of Alberta. After consenting to the study and providing their email address, patients self-reported their age, sex, gender identity, ethnicity, employment status, highest level of education, height, weight, and knee OA status, including prior use of non-surgical management strategies and interest in TKA. The first 3 digits of patients’ postal code was collected to distinguish rural and urban residency, also enabling post-hoc linkage with clinic A, B, or C invitation sharing as each had a distinct provincial jurisdiction coordinated through a centralized patient referral system.

### Intervention design

The 12-week digital intervention content was developed in partnership between the research team and healthcare professionals at Revive Wellness Inc (Edmonton, Alberta, Canada), including registered dietitians, psychologists, and kinesiologists. The intervention was based on accepted standards of practice for OA care, and included nutrition, mindfulness and self-care resources and components developed using behaviour change principles, described previously [18, 19]. Several new intervention resources (i.e. OA exercise videos) were developed specifically for this study, informed by prior research on exercises for knee OA [20, 21] while additional existing nutrition resources (e.g. goal setting worksheets, balanced meal plan guide) were used or adapted for patients with OA. The intervention content was delivered in two approaches:

- OA-specific content and resources were directly delivered to patients through weekly emails.
  ◦ Twelve unique weekly emails with knee OA specific content and attachments automatically delivered to patients every Monday morning for a 12-week period beginning the first Monday after the baseline questionnaires were completed. Emails were composed of a) nutrition recommendations including tips and meal planning guides, b) exercise instruction videos targeted for knee OA with progressions each week, and c) videos on mindfulness and advice regarding self-care, motivation, and stress management, including goal setting activities. A summary of the email content is provided in Supplementary information B.

b) Three additional components were available and could be accessed by patients on their own volition.
  ◦ Unlimited free access to a publicly available online subscription web-based platform [My Viva Plan©; discover.myvivaplan.com) designed to support general health and wellness behaviour change through nutrition, physical activity, and mindfulness supports. My Viva Plan© was developed by a registered dietitian, and platform resources support users to self-manage positive health behaviours through tracking, monitoring, and encouragement. This platform has been described elsewhere [19]. Access to the platform was provided to study patients through an enrollment link shared via email. The platform required new members to create an online profile and fill in personal information including medical history, food allergies, lifestyle behaviours, and current medications. Information on privacy and security of the platform was communicated to patients and they had the option to complete a profile.
  ◦ Free attendance at live online ‘Ask the Expert’ webinar sessions held the first and second-last Monday of each month. Patients could attend these sessions remotely through an internet-connected device, and attendance was not recorded. Patients signed in only with their first name and no video or microphone input was enabled. Webinar sessions had no specific content, but attendees could type in questions they had for the session leaders related to nutrition, exercise, or mindfulness. Health professionals led the session in pairs on a rotation schedule, and included registered dietitians, a registered psychologist, and a kinesiologist. Patients were invited by email with a link to attend six unique sessions during their 12-week intervention period.
  ◦ One free 30-min one-on-one personal phone or videoconference consult with a registered dietitian.

### Intervention delivery

Enrolled patients first completed all baseline outcome measure questionnaires remotely through REDCap at onset. Once questionnaires were submitted, they received an automatic email with an overview of the 12-week intervention and information about connecting with all components (including the directly delivered emails, and additionally accessible resources). Emails were sent out Monday morning of each week with the arthritis specific resources (outlined in Supplementary information B), and a second email was sent biweekly with information on the ask the expert webinars. Patients were encouraged to engage with the intervention resources at least three times per week, however there were no requirements or penalties for minimum interaction or attendance thresholds. Those who had not created a profile in the online web-platform after the first two-weeks were contacted by email and offered email or phone support to facilitate platform engagement.

### Outcomes

Acceptability of a healthcare intervention is determined by whether it meets the needs, preferences and expectations of the patients who receive it [22, 23]. For this study, acceptability was assessed by qualitative data from interviews. This includes aspects of the intervention that were stated as beneficial or enjoyable, and perceptions of information or support that was missing or desired. Engagement (i.e. use and adherence) is known to influence acceptability, particularly related to emotional and cognitive responses to intervention components [22]. Therefore, engagement with intervention resources was also explored by examining personal responses regarding engagement with the intervention from qualitative data. We focused on data reflecting intervention-level design or delivery factors, rather than personal-level factors (i.e. capability or motivation). This was purposeful to reflect aspects of the digital intervention design that could be adjusted or modified to support engagement in future interventions.

Quantitative data on participant retention at 12-weeks was also collected as an indication of acceptability. Web-platform-obtained data on weekly usage frequency was also collected. The number of individuals attending biweekly expert discussion sessions and requesting RD consultation was collected, however participant-level identification was not recorded to support anonymous attendance.

Preliminary effectiveness of the intervention was evaluated using a number of approaches, including change in health-related quality of life, well-being, mindfulness, and self-efficacy scores between baseline and 12-weeks. Health-related quality of life was measured with the 36-Item Short Form Health Survey (SF-36) [24] which evaluates eight different components, including physical functioning, role limitations due to physical health or emotional health, energy/fatigue, emotional well-being, social functioning, pain, and general health (each item scored from 0–100). Well-being was determined using the Warwick-Edinburgh Mental Wellbeing Scale (WEMWBS) [25], which uses a 5-item Likert scale over 14 questions (total score range of 14–60). The Five Facet Mindfulness Questionnaire (FFMQ) [26] assessed mindfulness in 5 domains with a 39-item questionnaire (total score range of 39–195). Self-efficacy for chronic disease management was assessed with the PROMIS Self-Efficacy for Managing Chronic Conditions Short Forms for Managing Daily Activities, and Managing Symptoms [27]. Each includes 8 questions about self-efficacy scored using a 5-item Likert scale (score range of 8–40). Arthritis-specific self-efficacy regarding pain, function, and other symptoms was assessed with the Arthritis Self-Efficacy Scale (score range of 1–10 for each domain) [28]. Additionally, change in self-report of knee OA severity, understanding of knee OA symptoms and treatment options, and interest in proceeding to knee replacement between baseline and 12-weeks were assessed. For severity, scoring was 1 = mild, 2 = moderate, or 3 = severe. Understanding of knee OA symptoms and treatment options was assessed using a four-item Likert scale [from 0 = not at all understanding, to 4 = completely understanding]. Interest in proceeding to have a knee replacement within the next 1 year was assessed with a yes/no question. Effectiveness of the intervention was also assessed by perceptions of positive health behaviors or health benefits as a result of receiving the intervention resources, determined from qualitative interview data.

### Study completion

At the end of the 12-week intervention, an email was automatically sent to patients with a link to complete the final online electronic questionnaires. All questionnaires were completed remotely through electronic links managed through REDCap. A total of four reminder emails were sent within two-weeks to promote final questionnaire completion. Study completion was identified by patients finishing these 12-week questionnaires.

Once final questionnaires were completed and submitted a subset of patients were invited by email for an optional interview via phone or videoconference. Invitations for interviews were shared by email, and participation was optional. All interviews were conducted by a member of the research team trained in qualitative methods (KG) and were audio-recorded and transcribed verbatim. The first 10 patients who completed the study were invited to participate in an interview. Purposeful sampling was used subsequently to invite a diverse representative pool of patients from different groups to complete an interview (i.e. male and female subjects, age < 65 and > 65 years, urban and rural residences, individuals who completed the 12-week intervention and individuals who notified of study withdrawal before 12-week completion). Interviews were conducted via phone or video-conference as selected by the participant. Interviews followed a semi-structured interview guide (Supplementary information C). Questions were organized to elicit perspectives on: a) aspects of the platform and information that they used and found beneficial, b) self-perception of health behavior changes made as a result of the intervention, c) enjoyment of intervention resources, d) barriers and facilitators of using the intervention resources, and e) perspectives on what additional information or support would be beneficial. Clarifying and elaborating probes were used to obtain additional in-depth answers as needed. Field notes were taken and interviews were audio-recorded upon consent and transcribed verbatim.

### Sample size

Sample size calculations for this study were based on anticipated acceptability completion rates of a minimum of 30 patients finishing the full 12-week program. With previous digital health interventions [29] reporting non-usage attrition of up to 83% of patients, we anticipated inviting up to 200 individuals to enroll to reach this threshold. Invitations were shared until *n* = 30 completed all study requirements.

### Statistical analyses

Normality of distribution in the quantitative data was tested using the Shapiro-Wilks test. Examination of change in scores from baseline to final (after 12-week intervention) were conducted using paired t-test or Wilcoxon signed rank test for non-parametric data. Differences in proportion were analyzed using Fishers exact test. All testing analyses were two-tailed, and a *p* value of < 0.05 was considered significant. Analyses were completed using IBM SPSS Statistics v28 (IBM Corp., Armonk, NY).

Interview transcripts were analyzed using content analysis. Qualitative description method was used to apply naturalistic inquiry to understand the participant’s lived experience in the context of their lives [30]. Data is gathered directly from individuals experiencing the phenomenon, capturing their words to identify socially constructed health needs and inform clinical care practices through an inductive process [31, 32]. Two members of the research team (KG) and (MQ) were responsible for independently coding transcripts and bringing emerging categories to the research team for review, discussion, and verification. Data were organized into codes and then integrated into broader categories to inductively generate answers to the research questions as described by patients’ experiences. No theoretical framework was applied in our data analysis. Codes determined directly from the data were identified by initial line-by-line reading of the first seven transcripts, followed by consensus between the two coders. A codebook was developed and used to complete the analysis of the remaining interview transcripts. New codes identified in the remaining interview transcripts were added to the codebook. Interview transcripts were not returned to patients, nor were any additional follow-up interviews conducted. Interview data was coded manually and managed using NVivo 12 (QSR International). Findings for the qualitative data are reported using the Consolidated Criteria for Reporting Qualitative Research (COREQ) checklist [33].

## Results

**Fig. 1:**
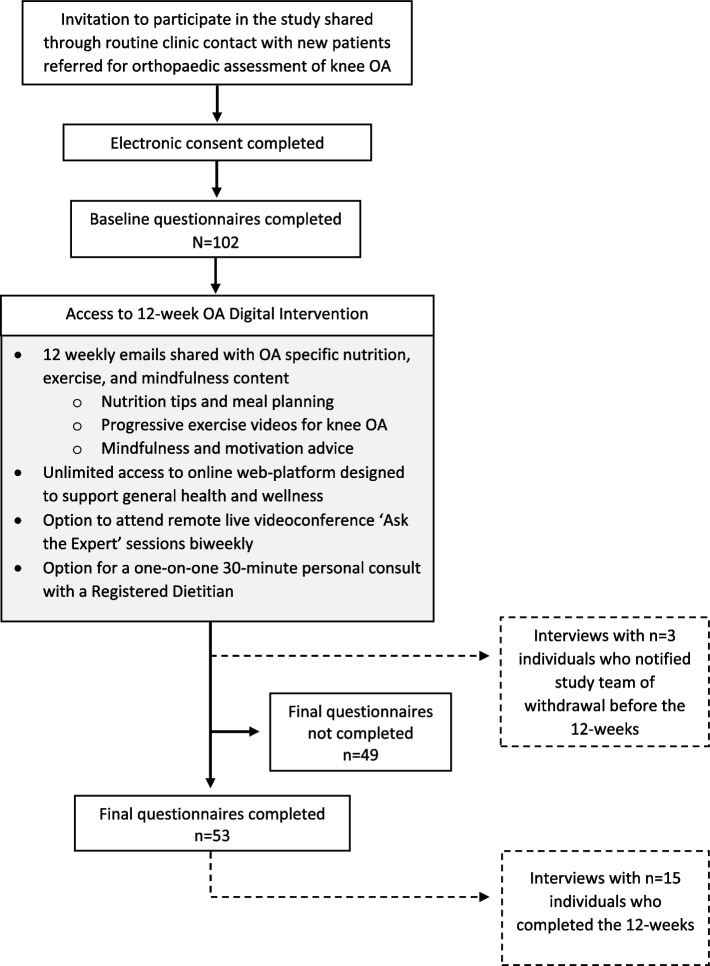
Participant flow through the study

Participant flow through the study is outlined in Fig. 1. Invitations to self-enroll were shared through the clinics from Oct. 5, 2020 until May 31, 2021. A total of *N* = 102 individuals self-enrolled and completed all baseline questionnaires (73.5% female, age 64 ± 7 years, body mass index 32.9 ± 7.3 kg/m^2^), with *n* = 53 (52.0%) finishing all 12-weeks and final questionnaires. Patients who completed the 12-week study had a mean age 65 ± 7 years (range 48–83 years); mean BMI 33.4 ± 6.3 kg/m^2^ (range 25.5–45.8 kg/m^2^), and 77.4% resided in an urban centre (Table 1). The majority had bilateral OA (69.8%), with 71.7% self-identifying their knee OA as severe at baseline. Posthoc examination of postal code data indicated that all patients who enrolled received their invitation from clinic A. A description of study non-completers is provided in Supplementary information D. Patients who completed the study did not appear to be different than the non-completing group, except in the proportion working full-time (67.9% vs. 26.5%, respectively).

**Table 1:** Demographics of patients who completed the 12-week intervention (*n* = 53)

	**Total** ***n* = 53**	**Females** ***n* = 38, 71.7%**	**Males** ***n* = 15, 28.3%**
Age (years), mean (SD)	65.0 (6.78)	64.9 (7.2)	65.2 (5.8)
Height (cm), mean (SD)	166.8 10.3)	162.0 (7.0)	179.1 (6.2)
Weight (kg), mean (SD)	93.1 (19.5)	91.1 (18.8)	98.1 (20.7)
BMI (kg/m^2^), mean (SD)	33.4 (6.3)	34.6 (6.1)	30.5 (6.1)
Ethnicity, White, n (%)	46 (86.8)	32 (84.2)	14 (93.3)
Education > high school, n (%)	41 (77.4)	29 (76.3)	12 (80.0)
Employed, full time, n (%)	36 (67.9)	23 (60.5)	13 (86.7)
Reside in rural area, n (%)	12 (22.6)	7 (18.4)	5 (33.3)
Bilateral knee OA, n (%)	37 (69.8)	26 (68.4)	11 (73.3)
Self-report knee OA as severe at baseline, n (%)	38 (71.7)	27 (71.1)	11 (73.3)
Symptomatic duration > 5 years, n (%)	35 (66.1)	25 (65.7)	10 (66.7)
Previous treatments used to manage knee OA, n (%):			
Physiotherapy	33 (62.3)	25 (65.8)	8 (53.3)
Exercise program	46 (86.8)	31 (81.6)	15 (100.0)
Arthritis-specific exercise program (i.e. GLAD)	8 (15.1)	6 (15.8)	2 (13.3)
Injections	37 (69.8)	26 (68.4)	11 (73.3)
Brace	24 (45.3)	18 (47.4)	6 (40.0)
Orthotics	13 (24.5)	9 (23.7)	4 (26.7)
Registered Dietitian	7 (13.2)	4 (10.5)	3 (20.0)
Prescription pain medications	23 (43.4)	14 (36.8)	9 (60.0)
Over the counter pain medications	49 (92.5)	35 (92.1)	14 (93.3)

*BMI* Body mass index, *OA* Osteoarthritis, *SD* Standard deviation

Data from *n* = 18 interviews were collected (transcribed data from 17 audio-recorded interviews, and written data from 1 interview with an individual who was hearing impaired). Of the first 10 individuals to complete the study, 8 agreed to participant in an interview. Purposeful sampling enabled an additional 7 interviews with study completers, plus 3 interviews with individuals who withdrew before the study end (Fig. 1).

### Acceptability

Acceptability of the intervention was identified by primary positive perspectives related to acceptability in interview data. Acceptability was connected to two themes: 1) tailored and reliable information, and 2) preferences for online or offline content.

The intervention resources, particularly email content, were positively perceived as curated and designed specifically for individuals with knee OA. Having reliable, tailored information delivered directly to them was valued by patients who acknowledged the challenge and effort of filtering health information from the internet to their specific needs.

*“It’s better than googling, looking for information. If you google it, you’d get lots, maybe too many. But with you specifically sending the videos and links, nutrition stuff, you as a doctor choose for us, and that’s much better.” (Participant D)*

*“I do go on website [My Viva Platform] and look at things and everything but I didn’t know what to expect and then the emails started. So I just stayed with the emails and what they were doing, which was okay. I liked it. I enjoyed it.” (Participant M)*

Patients described that the digital intervention resources made them feel less isolated in their journey managing their OA, and in more control of their health.

*“Because with the resources that you’re giving me, you’re sending to me, I feel that I’m not alone. So there’s someone just going to in this way, I’m not the only one who has this. At least I have a little confidence that this is not really the end of my world. There is somebody who can help me to really boost my morale.” (Participant B)*

Individuals’ preferences for online versus offline intervention resources were connected with acceptability. Although most described the digital delivery process as acceptable, some made adaptations or noted preferences for offline resources or connecting in-person with a health care provider.

*“Instead of watching them [videos] online all the time, I watched them a couple of times, but then I would write them down. And then I’d write a little blurb at the bottom on how to do it. And then that’s what I would follow. I’m a paper person, right?” (Participant I)*

*“I think for me, with the exercise, it’s nice to have a coach or to have somebody who checks up on you once a week or once every two weeks and says, ‘Hey, how are you doing? Let’s look at your calendar. How many days did you go? How many days did you miss? How much time did you do? Keep on going. Keep up the good work.’ ”(Participant K)*

A technology-related finding described by some patients was related to input and storage of personal information on the web-platform. Not all patients were comfortable with the perceived personal questions and amount of information collected on the online platform.

*“[Web-platform] I did not care for it all. I felt it was too… I don't know. Just something's not quite right about it. This was dealing with my arthritis, my osteoarthritis. But when I go onto the website, I felt like the website was digging too personal into your lifestyle, like if you're going walking or reading-- it just seemed like it was too personal.” (Participant M)*

#### Engagement

Engagement with the resources was both positively and negatively influenced by intervention-level design and delivery factors. The majority of participant responses identified positively with the exercise videos. The personalization to knee OA and the body size and age of the person demonstrating exercises was relatable, making patients more comfortable engaging with the exercises.

*“I’m emphasizing this now, exercise videos are the best. And putting an older guy and obviously overweight, that’s best. Tiny, slim kind of girl model…I wouldn’t be motivated to do that. But because the guy was doing it, I thought, ‘I can do it if he’s doing it’.” (Participant D)*

One aspect that both supported and hindered engagement was having content directly delivered each week through emails, as exemplified in the following contrasting quotes.

*“If I could have you guys sending me [emails] every week to do that, I’d probably do exercises every week. Now I have to remind myself to do it myself.” (Participant I)*

*“It actually felt a lot of times like it was unfinished homework.” (Participant C)*

Patients who withdrew from the study noted a need for more advanced-level exercises, or options to further personalize the nutrition intervention.

*“What I need is a personal touch of more variety and the next level. I mean, I may be a senior but I want the expectation of everything.” (Participant M)*

*“I think that the program could be very good for a lot of people, and I think online as a whole – because there’s lots of people who can’t get out to a gym or can’t afford a gym – it’s a good idea. It’s just not for where I’m at and what my diet is today, what my exercise is today, this just wasn’t the right program for me.” (Participant J)*

Some patients described that the nutrition information, especially menu ideas, may not have fit their habits and preferences, making them difficult to implement. Interestingly, patients views on the menus were very diverse – *“more for vegetarians” (Participant M), “quite exotic” (Participant Q), “don’t list the sugars” (Participant J)* – and demonstrated that when tailoring wasn’t perceived by patients, there was less engagement with resources.

Perceptions that nutrition resources were designed to support weight change may also have negatively shaped patients openness to engage with nutrition components.

*“I thought they [nutrition resources] were more focused on weight loss.” (Participant K)*

*“My weight is not bad. I think I was on the borderline for registering for this program. And so I felt that I can manage my weight, and I know how to eat well.” (Participant Q)*

Study completion rate was 52%. Only a small proportion of patients (*n* = 7, 13.2%) interacted with the online web-platform 3 or more times/week during the study (Supplementary information E). A greater proportion of patients either did not engage at all with the web-platform (*n* = 8, 15.1%), or engaged infrequently [≤ 5 times in total over the 12-week study] (*n* = 25, 47.2%). Additionally, between 1–6 patients logged-in to each live webinar session and engaged in written questions with the session leaders. No study patients arranged for a one-on-one meeting with a dietitian.

### Preliminary effectiveness

Improvements in health-related quality of life components of pain and physical functioning were identified after the 12-week intervention through quantitative data [mean increase in SF36 subitem scores of 4.4 (*p* = 0.016) and 6.7 (*p* < 0.001), respectively], along with improved self-efficacy for managing symptoms and managing daily activities [mean increase in PROMIS T-scores of 2.2 (*p* = 0.003) and 4.4 (*p* < 0.001), respectively] (Table 2). There were also higher Likert scores regarding understanding of arthritis symptoms after the intervention [mean increase of 0.3 (*p* < 0.001)], and a 1.8% reduction in interest to proceed to a TKA (Table 2). No change in mindfulness or well-being were identified between baseline and after the intervention. A 17% reduction in self-perception of severe knee OA was noted after the intervention, however this was not statistically significant.

**Table 2:** Change in health-related quality of life, well-being, mindfulness, self-efficacy, severity and understanding of arthritis, and interest in TKA after the 12-week intervention (*n* = 53)

	Baseline	Final	Mean difference (95% CI)
SF-36			
Physical Functioning	33.0 (21.5)	39.7 (24.0)	6.7 (2.7 – 10.7) **
Role limitations due to physical health	24.5 (35.5)	32.1 (37.8)	7.5 (-2.9 – 18.0)
Role limitations due to emotional health	51.6 (46.5)	47.8 (43.6)	-3.8 (-16.7 – 9.2)
Energy/fatigue	46.9 (20.1)	47.5 (21.1)	0.7 (-3.3 – 4.6)
Emotional well-being	71.0 (17.6)	72.0 (17.6)	1.0 (-1.7 – 3.7)
Social functioning	67.7 (23.2)	70.8 (20.9)	3.2 (-2.1 – 8.5)
Pain	35.7 (18.3)	40.1 (18.8)	4.4 (0.2 – 8.6) *
General health	59.6 (20.9)	61.5 (19.4)	1.9 (-1.6 – 5.4)
Warwick Mental Well-being	50.3 (10.1)	50.1 (9.6)	-0.1 (-1.9 – 1.6)
Five Facet Mindfulness			
Total	141.5 (19.7)	143.0 (18.6)	1.5 (-2.0 – 5.1)
Observing	29.7 (5.2)	30.1 (4.3)	0.4 (-0.7 – 1.6)
Describing	28.5 (6.3)	28.1 (5.8)	-0.3 (-1.4 – 0.7)
Awareness	29.8 (6.2)	30.0 (6.0)	0.2 (-0.7 – 1.2)
Non-judgemental	29.4 (5.8)	30.4 (5.9)	1.0 (-0.3 – 2.2)
Non-reactivity	24.1 (4.5)	24.4 (4.8)	0.2 (-0.9 – 1.4)
PROMIS Self-Efficacy for Managing Symptoms			
Raw score	24.7 (7.1)	26.9 (7.3)	2.2 (0.8 – 3.6) *
T-score	42.1 (6.9)	44.2 (7.4)	2.2 (0.8 – 3.5) *
PROMIS Self-Efficacy for Managing Daily Activities			
Raw score	25.3 (6.6)	29.4 (7.4)	4.1 (2.7 – 5.5) **
T-score	41.3 (4.7)	45.7 (7.9)	4.4 (2.8 – 6.0) **
Arthritis Self-Efficacy Scale			
Pain	5.4 (2.1)	5.5 (2.2)	0.1 (-0.3 – 0.5)
Function	6.9 (1.8)	7.1 (1.9)	0.2 (-0.2 – 0.6)
Other Symptoms	5.9 (1.9)	6.3 (2.0)	0.4 (-0.1 – 0.8)
Self-reported knee OA severity, n (%)			
Mild	0 (0)	2 (3.8)	3.8%^a^
Moderate	15 (28.3)	22 (41.5)	13.2%^a^
Severe	38 (71.7)	29 (54.7)	-17.0%^a^
Understanding of arthritis	2.92 (0.94)	3.28 (0.79)	0.3 (0.1 – 0.6) **
Interest in a TKA within next year, n (%)	42 (79.2%)	41 (77.4%)	-1.8%^a^ **

Values presented are mean (SD) unless otherwise indicated

Scoring parameters (min, max) for each measure: SF-36 0–100; Warwick 14–60; Five Facet Mindfulness Total 39–195, Domains 8–40, except Non-reactivity 7–35; PROMIS Raw Scores 8–40; Arthritis Self Efficacy 1–10; Understanding of arthritis 0 (not at all) – 4 (completely)

*SF-36* 36-item short form survey, *TKA* Total knee arthroplasty, *PROMIS* Patient-Reported Outcomes Measurement Information System

^a^ Difference in proportion

^*^p < 0.05

^**^p < 0.001

Effectiveness of the intervention was also identified by perspectives of perceived benefits from qualitative interviews. Patients commonly described that by exercising more regularly and appropriately using exercises designed for their OA, and/or changing habits linked to the nutrition or self-care resources, they experienced benefits related to their OA and overall health.

*“It’s [intervention] very helpful for me because I have resources to use on how I will minimize the pain. I have the resources to use of what kind of exercises I need to do to strengthen my… because the pain is still there, it’s not going to be gone but at least to strengthen my legs.” (Participant B)*

*“I didn’t realize the effect of lack of sleep had on the metabolism and that type of thing. So that has really helped me. If I’m tired, I go to bed and I sleep whereas before I’d try to tough it out, but I don’t anymore.” (Participant A)*

*“I think following those exercise routines really does help with my arthritic pain. If I don’t do those exercises, I really tell the difference.” (Participant K)*

*“It’s [the study intervention] influenced 80% on my overall health. Because when I started the diet…I don’t know if it’s in relation to the diet I’m taking but during how many weeks I’m doing the diet, when I went to the laboratory I can see the cholesterol part is lower down and my sugars because I’m doing a bit of exercise in combination with the diet. So I can see if I continue this part, not only my knee will benefit, (but) also my health.” (Participant B)*

In some individuals, this resulted in rethinking OA treatment plans.

*“I noticed quite a bit of improvement. And I finally got a call from the specialist for a knee replacement, and I said I didn’t want the knee replacement yet because I found these exercises – I told him I was doing these exercises and I thought, ‘they feel great.’ I can walk more. I can do quite a few different things.” (Participant I)*

In contrast, a participant described that their existing knee pain, in addition to their perception that only the surgery could help them, might have negatively influenced their perspective on the study resources and, as a result, the benefits they experienced from participating in the study.

*“I will be getting a knee replacement soon so maybe the stage of illness I was in affected my loss of interest. For me what would probably be helpful is information about the surgery, tools to help with recovery.” (Participant N)*

## Discussion

**Fig. 2:**
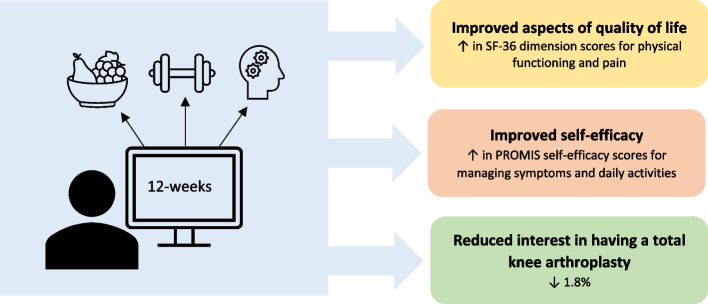
A 12-week digital nutrition, exercise, and mindfulness intervention for patients with advanced knee OA showed preliminary effectiveness to improve patient outcomes and OA self-management. SF-36 = 36-item short form survey, PROMIS = Patient-Reported Outcomes Measurement Information System

In individuals with advanced knee OA, a 12-week remotely delivered digital intervention was acceptable to patients and showed preliminary effectiveness for improving physical functioning and pain-related quality of life, and self-efficacy for managing their chronic disease (Fig. 2). Further, there was a 17% reduction in self-perception of severe knee OA, and a 1.8% reduction in interest in proceeding to a knee replacement following the intervention. These findings suggest that remote digital interventions could have meaningful implications on patient self-management of OA and clinical outcomes, and could potentially decrease use of surgical health care resources. This warrants further investigation.

Improvements in self-efficacy related to management of daily activities appeared to reach clinically relevant thresholds after the intervention (mean change 4.4, 95% CI 2.8 – 6.0). The literature supports that a PROMIS T-score change above 3 is important [34]. Improvements in self-efficacy can have positive impacts on how individuals continue to live with and manage their knee OA. Prior studies have identified that increasing mobility-related self-efficacy can reduce pain levels and improve physical function [35], which could potentially enable individuals to postpone or prevent the need for more intensive interventions such as arthroplasty.

Our findings were similar to a previous randomized clinical trial (RCT) examining a 12-week digital intervention for knee OA in subjects from the United States [36], which also found improvements in understanding of OA and decreased interest in proceeding to TKA after the intervention. Another RCT examined digital reminders to complete exercises for knee OA [37]. This study noted greater improvements in pain and function in the group who received notifications, indicating that remote connection with patients may support outcome improvement [37]. An RCT by Bennell et al. [38] found that telehealth video-based delivery of a nutrition and exercise intervention for OA was more effective than a web-based education control, which suggests that the addition of personalized connection with a health care provider (even remotely) could enhance the effectiveness of a digital intervention. This is of interest considering our qualitative findings with respect to the nutrition component, where patients negative perceptions or predispositions around nutrition advice may have impacted their interaction with nutrition resources. While the addition of facilitation or interpretation assistance by a health professional at onset could potentially improve the personalization and engagement with the digital nutrition resources, it is noteworthy that this option was available in the current study, however, no patients requested the free consult with the registered dietitian. This may have been a communication issue, requiring more explicit information about the availability and potential benefits of a consult. There may also be underlying barriers related to patients’ perceptions of dietitians as information and education providers only, rather than providing more individualized therapeutic and counselling support [39].

The importance of nutrition for OA may also need greater emphasis for this patient population. We found engagement and acceptability of nutrition resources was mixed, with fewer positive comments related to this intervention component. This appears linked to inaccurate perceptions that nutrition is synonymous with weight loss or diet, despite our design to purposefully exclude any information to this respect in the intervention resources or the web-platform settings. However, these types of perceptions may still be interpreted by patients, potentially supported by pervasive societal diet culture or advice from healthcare providers regarding the benefit of weight reduction for OA [40].

Changing communication and education strategies to disentangle presumptions about nutrition and weight loss could be beneficial. This could be done by highlighting the value of nutrition for supporting muscle mass preservation and decreasing inflammation-related metabolic factors that are both very relevant for effective OA management. Further, broadly developed nutrition resources (e.g. meal planning) may need to include customizable options for different dietary patterns.

The timing of delivery of this digital intervention to individuals waiting for an orthopaedic consult was received positively by many patients, however some felt their OA was too advanced at this stage and felt that surgery (TKA) was the only option. This could suggest that digital interventions provided earlier in the OA disease process may be preferential in some individuals. Alternatively, this could also reflect patient assumptions about the OA disease trajectory [41], and including information to the digital intervention on TKA expectations and satisfaction could potentially change perceptions on the inevitability of joint replacement [42].

A unique finding from this study was related to concerns around privacy and personal information collected in external digital health platforms. These concerns may influence participant’s perceptions of acceptability and engagement with digital health. Patient education may be a potential solution, with a clear explanation at onset of how personal information will be used to customize the digital health experience. However, questions about necessity of information collected, immortality of data, and patients’ uncertainty with how some personal data could be interpreted are legitimate issues in the digital health space that need to be examined further in future investigations [43].

Study completion rate was 52%, which suggests moderate acceptability considering that dropout rates can be high (i.e. 72–83%) in electronic health interventions, especially for chronic disease management [29]. However, retention of participants in this digital intervention may be further improved by addressing privacy concerns, nutrition-related communication, and through patient-engagement to identify potential retention barriers.

### Strengths and limitations

This study was conducted during the covid-19 pandemic in an environment where there was limited access to in-person health care services, a stoppage of arthroplasty surgery, and no access to indoor community recreation activities. As a result, the remotely delivered digital intervention approach was a timely strength, providing patients with access to resources when they were isolated at home. These circumstances could also be a limitation, and study findings may differ if pandemic-related restrictions and associated stressors were not in effect. All individuals who participated in the study were inadvertently engaged only through one clinic site, despite efforts to enroll through three clinics with consistent information distributed across sites. This suggests that clinic processes and relationships with patients may have strong impacts on participation and engagement in a pragmatically designed study and should be considered in future research. Notably, the development of OA specific content for the digital intervention was another key strength, supporting that digital health platforms should be willing to adapt and expand to address health conditions of patients. Interestingly, the patients who did or did not complete the study do not appear to be different, except with respect to the proportion who were working full-time (67.9% of study completers vs. 26.5% of non-completers). This may suggest that individuals who are no longer in the workforce may need different approaches to engage them in digital care, which should be explored in future studies. This study included a variety of intervention delivery approaches (email, web-based platform, webinars), preventing us from clearly identifying which aspects were primarily responsible for impacting change in outcomes. Notably, our sample size calculation did not consider effectiveness so the study may have been underpowered for this analysis. Further, there was no comparison to a control group, and positive outcome changes may have been due to non-specific effects or regression to the mean. There are also limitations with the multiplicity of analyses and inclusion of only study completers in the final analyses. The study was not prospectively registered. Future studies, including randomized controlled trials, would provide clearer comparisons and rigorous assessment of intervention effectiveness and changes in behavioural outcomes such as macronutrient intake and exercise frequency. These limitations may reduce the generalizability of the findings. Additional research will be needed prior to clinical implementation.

## Conclusion

A single-arm 12-week multimodal digital nutrition, exercise, and mindfulness intervention for knee OA was acceptable to patients and showed preliminary effectiveness in improving self-efficacy for chronic disease management, and aspects of quality of life related to pain and physical functioning. Remotely delivered digital health interventions may be a pragmatic and acceptable approach to improve patient outcomes and OA self-management.

## Supplementary Information

**Additional file 1: Supplementary Information A.** Sample invitation to participate. **Supplementary Information B.** Content of weekly emailed intervention resources. **Supplementary Information C.** Semi-structured interview guide. **Supplementary information D.** Description of *n*=49 individuals who enrolled but did not complete the 12-week study. **Supplementary Information E.** Weekly access frequency with the online web-platform in *n*=53 individuals who completed the 12-week study.

## Data Availability

The datasets generated and analysed during the current study are not publicly available due to confidentiality agreements with participants. For questions, please contact the corresponding author.
