# Supplementary material for: Acceptability and preliminary effectiveness of a single-arm 12-week digital behavioral health intervention in patients with knee osteoarthritis
Source: BMC Musculoskelet Disord. 2023 Feb 17;24:129. doi: 10.1186/s12891-023-06238-8 (PMC9936108; doi:10.1186/s12891-023-06238-8)
Supplement: Supplementary file 1 — Additional file 1: Supplementary Information A. Sample invitation to participate. Supplementary Information B. Content of weekly emailed intervention resources. Supplementary Information C. Semi-structured interview guide. Supplementary information D. Description of n=49 individuals who enrolled but did not complete the 12-week study. Supplementary Information E. Weekly access frequency with the online web-platform in n=53 individuals who completed the 12-week study. [file 12891_2023_6238_MOESM1_ESM.docx]

**Supplementary Information A**

***Sample invitation to participate***

| **You are invited to participate in a research study**  *Help us improve knee arthritis care*   - Nutrition, physical activity and self-care can have positive impacts on the symptoms of knee arthritis, and help improve overall health and quality of life. - However, access to in-person support in these areas is currently limited for individuals with knee arthritis. - We would like to know if remotely-delivered online support would be beneficial, and if patients would enjoy this type of care.   This research study will evaluate the benefits and patient-experience of using an online nutrition and wellness platform and resources for a 12-week period.  Adults who have been referred to [clinic name] for assessment of their knee arthritis can choose to participate. There is no obligation for you to be involved in this study if you are not interested, and participation will not influence your care in the clinic or access to surgery.  **If you decide to participate, you will receive free access for a 12-week period to an online wellness platform, receive weekly education resources shared through email, and have the option to attend live bimonthly webinars led by health professionals.**  To enroll, you will need to complete an online survey and questionnaires at the beginning and end of the 12-week period. We ask you to interact and use the online resources at least 3 times per week. You may be invited to a phone interview at the end of the study to share your thoughts and experiences of using this online support.  **To enroll or learn more, use the following link to access the study information page:**  **[URL]**  **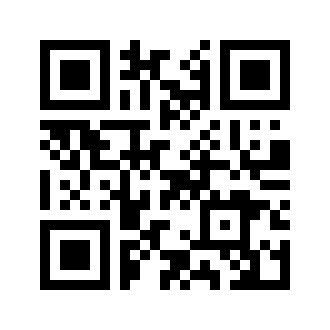**  **You can also contact the research team by email to ask any questions [email address]**  *This project is led by a research team from the University of Alberta, and funded with support from Alberta Health Services through the Bone and Joint Health Strategic Clinical Network* |
| --- |

**Supplementary Information B**

***Content of weekly emailed intervention resources***

| **Week** | **Summary information, printable handouts, and videos provided through email** |
| --- | --- |
| 1 | - Week 1 overview sheet - Printable: My Why for Health (goal setting worksheet) - Video: Power of Gratitude - Printable: Physical activity guidelines - Exercise video 1: seated isometric leg press; seated isometric hamstring curl; seated isometric hip abduction |
| 2 | - Week 2 overview sheet - Printable: Building a Balanced Breakfast and Better Breakfast Scenarios - Video: Breakfast Basics - Printable: Action Plan for Goal Setting - Exercise video 2: seated knee extension; seated hamstring curl; seated hip abduction (with resistance bands) |
| 3 | - Week 3 overview sheet - Printable: Lunch Basics and Building a Balanced Lunch and Dinner - Videos: Lunch Basics - Exercise video 3: seated knee extension; seated hamstring curl; seated hip abduction; sit to stand; seated calf raises |
| 4 | - Week 4 overview sheet - Printable: Healthy Meal Hacks and Dinner Basics - Video: Dinner Basics - Printable: 4 Ways to Fight Aches & Pains at Work: Sitting - Exercise video 4: sit to stand; standing hamstring curl; step-ups; seated calf raise; standing hip abduction (bent leg) |
| 5 | - Week 5 overview sheet - Printable: Building a Balanced Snack - Video: The Power of Snacking - Blog: How Often Should We Work Out? - Exercise video 5: split stance sit to stand; standing hamstring curl; step-ups; standing calf raises; standing hip abduction (bent leg) |
| 6 | - Week 6 overview sheet - Printable: Sleep Hygiene - Video: Tuck Me In – A Pre-Sleep Meditation - Video: The Importance of Sleep - Exercise video 6: split stance sit to stand; standing hamstring curl; split stance isometric lunge hold; bilateral seated calf raise from drop; standing hip abduction (straight leg); standing hip adduction with resistance band |
| 7 | - Week 7 overview sheet - Printable: Energized Eating - Video: MVP Weekly Coaching Series – Power of Nutrition - Blog: Know Your Fitness Limits - Exercise video 7: split stance sit to stand; hip hinge (dumbbell deadlift); split stance isometric lunge hold; bilateral standing calf raise from drop; standing hip abduction (straight leg); standing hip adduction with resistance band |
| 8 | - Week 8 overview sheet - Printable: Meditation 101 - Video: Meditation of Breath, and Intro to My Viva Yoga - Video: Mentally Fit - Exercise video 8: split stance sit to stand; hip hinge (dumbbell deadlift); reverse lunge with slider; bilateral standing calf raise with drop; standing clamshells; lateral lunge with slider |
| 9 | - Week 9 overview sheet - Printable: The Science of Stress - Video: How Stress Affects Taking Time for You - Blog: Potential Benefits of Seeing a Psychologist Online - Exercise video 9: partial wall squats with or without exercise ball; hip hinge (dumbbell deadlift); reverse lunge with slider; standing calf raise with drop; standing clamshell; lateral lunge with slider |
| 10 | - Week 10 overview sheet - Printable: Finding Balance - Video: Stress: How it Affects you Physically - Blog: Workout Recovery Explained - Exercise video 10: partial wall squats with or without exercise ball; hip hinge (dumbbell deadlift); forward step-down touches (single leg partial squat); standing calf raise with drop; lateral monster walk with thigh band; lateral lunge with slider |
| 11 | - Week 11 overview sheet - Video: MVP Coaching Series - Treats - Printable: Including Treats in Your Routine, Including Treats in Your Routine Worksheet, Understanding Cravings - Video: Exercise progression advice - Exercise video 11: squats; hip hinge (dumbbell deadlift); forward step-down touches (single leg partial squat); standing calf raise with drop (one leg eccentric); lateral monster walk with thigh band; lateral lunge with slider; |
| 12 | - Week 12 overview sheet - Video: MVP Coaching Series – Patience - Self Reflection - Printable: Motivation and Goal Setting - Video: Reflection in Fitness - Exercise video 12: squats; hip hinge (dumbbell deadlift); forward step-down touches (single leg partial squat); standing calf raise with drop; lateral monster walk with thigh band; lateral lunge with slider |

**Supplementary Information C**

***Semi-structured interview guide***

| 1. Can you tell me about your overall experience in using the study resources over the past 12-weeks? |
| --- |
| 2. Information in the study was provided in three approaches: a) weekly emails, b) online digital platform, and c) live information sessions twice a month. Can you tell me about which aspects you used? |
| 3. What did you like best, or find most useful? (about any of these approaches) |
| 4. What did you like least, or find less useful? (about any of these approaches) |
| 5. How confident were you in accessing these approaches, or following suggestions that were provided? |
| 6. Did you know about or consider the option to meet for 30 minutes for free with a Registered Dietitian? |
| 7. Has your nutrition, activity, or other arthritis management health behaviours changed as a result of the information that was shared? |
| 8. Do you feel that this study experience had an influence on your knee arthritis or overall health? |
| 9. What would you change about the approach or information provided? Were there any aspects that were more or less challenging to use? |
| 10. What do you feel would make online care for knee arthritis more effective, easier to use, or more appealing to patients? |
| 11. Do you feel that access to an online/virtual program such as this would be beneficial for everyone with knee arthritis? |
| 12. Is there anything else you would like to share or comment on? |

**Supplementary information D.** Description of n=49 individuals who enrolled but did not complete the 12-week study

| Female subjects, n (%) | 38 (77.6) |
| --- | --- |
| Age (years), mean (SD) | 64.1 (7.3) |
| Height (cm), mean (SD) | 166.9 (9.6) |
| Weight (kg), mean (SD) | 90.5 (25.4) |
| BMI (kg/m^2^), mean (SD) | 32.5 (9.3) |
| Ethnicity, White, n (%) | 43 (87.8) |
| Education > high school, n (%) | 35 (71.4) |
| Employed, full time, n (%) | 13 (26.5) |
| Reside in rural area, n (%) | 14 (28.6) |
| Bilateral knee OA, n (%) | 38 (77.6) |
| Self-report knee OA as severe at baseline, n (%) | 36 (73.5) |
| Symptomatic duration > 5 years, n (%) | 36 (73.5) |

**Supplementary Information E.** Weekly access frequency with the online web-platform in n=53 individuals who completed the 12-week study

|  | **Frequency of Web-Platform Access/Week of Intervention** | | | | | | | | | | | | **Accessed Platform ≥3x/Week** |
| --- | --- | --- | --- | --- | --- | --- | --- | --- | --- | --- | --- | --- | --- |
| **Participant** | **1** | **2** | **3** | **4** | **5** | **6** | **7** | **8** | **9** | **10** | **11** | **12** | **-** |
| **1** | 14 | 41 | 40 | 25 | 44 | 32 | 28 | 45 | 40 | 26 | 8 | 41 | yes |
| **2** | 2 | 0 | 0 | 0 | 0 | 0 | 0 | 0 | 0 | 0 | 0 | 0 | - |
| **3** | 2 | 0 | 0 | 0 | 0 | 0 | 0 | 0 | 0 | 0 | 0 | 0 | - |
| **4** | 2 | 0 | 0 | 0 | 0 | 0 | 0 | 0 | 0 | 0 | 0 | 0 | - |
| **5** | 0 | 0 | 0 | 0 | 0 | 0 | 0 | 0 | 0 | 0 | 0 | 0 | - |
| **6** | 5 | 5 | 7 | 2 | 7 | 5 | 4 | 4 | 3 | 3 | 3 | 4 | yes |
| **7** | 3 | 11 | 5 | 2 | 10 | 4 | 1 | 3 | 3 | 2 | 1 | 1 | yes |
| **8** | 1 | 1 | 36 | 21 | 23 | 19 | 12 | 9 | 11 | 8 | 9 | 10 | yes |
| **9** | 1 | 4 | 1 | 3 | 0 | 0 | 0 | 0 | 0 | 0 | 0 | 0 | - |
| **10** | 2 | 0 | 1 | 0 | 0 | 0 | 0 | 0 | 0 | 0 | 0 | 0 | - |
| **11** | 1 | 0 | 1 | 0 | 0 | 0 | 3 | 8 | 5 | 0 | 0 | 0 | - |
| **12** | 5 | 2 | 9 | 4 | 3 | 6 | 1 | 0 | 0 | 0 | 0 | 0 | - |
| **13** | 0 | 0 | 0 | 2 | 0 | 0 | 0 | 0 | 0 | 0 | 0 | 0 | - |
| **14** | 1 | 2 | 1 | 0 | 0 | 0 | 1 | 0 | 0 | 0 | 0 | 0 | - |
| **15** | 1 | 0 | 0 | 0 | 0 | 0 | 0 | 0 | 0 | 0 | 0 | 0 | - |
| **16** | 6 | 1 | 3 | 0 | 0 | 0 | 0 | 0 | 0 | 0 | 0 | 0 | - |
| **17** | 1 | 0 | 0 | 0 | 1 | 3 | 0 | 0 | 0 | 0 | 0 | 0 | - |
| **18** | 3 | 0 | 0 | 0 | 0 | 0 | 0 | 0 | 0 | 0 | 0 | 0 | - |
| **19** | 5 | 3 | 4 | 5 | 3 | 0 | 0 | 0 | 0 | 0 | 0 | 0 | - |
| **20** | 2 | 1 | 2 | 0 | 0 | 0 | 0 | 0 | 0 | 0 | 0 | 0 | - |
| **21** | 33 | 44 | 27 | 36 | 43 | 45 | 29 | 24 | 12 | 2 | 2 | 8 | yes |
| **22** | 12 | 14 | 8 | 12 | 13 | 11 | 6 | 5 | 9 | 4 | 2 | 2 | yes |
| **23** | 0 | 0 | 0 | 0 | 0 | 0 | 0 | 0 | 0 | 0 | 0 | 0 | - |
| **24** | 0 | 0 | 3 | 4 | 4 | 3 | 1 | 0 | 0 | 0 | 0 | 0 | - |
| **25** | 2 | 2 | 0 | 0 | 0 | 0 | 0 | 0 | 0 | 0 | 0 | 0 | - |
| **26** | 4 | 6 | 3 | 5 | 6 | 7 | 4 | 6 | 4 | 6 | 6 | 4 | - |
| **27** | 5 | 7 | 0 | 0 | 0 | 0 | 0 | 0 | 0 | 0 | 0 | 0 | - |
| **28** | 4 | 2 | 3 | 2 | 0 | 0 | 0 | 0 | 0 | 1 | 0 | 1 | - |
| **29** | 1 | 0 | 0 | 0 | 0 | 0 | 0 | 0 | 0 | 0 | 0 | 0 | - |
| **30** | 4 | 0 | 0 | 0 | 0 | 0 | 0 | 1 | 0 | 1 | 0 | 3 | - |
| **31** | 6 | 18 | 11 | 22 | 12 | 6 | 7 | 7 | 6 | 7 | 7 | 6 | yes |
| **32** | 0 | 0 | 0 | 0 | 0 | 0 | 0 | 0 | 0 | 0 | 0 | 0 | - |
| **33** | 1 | 3 | 1 | 1 | 0 | 0 | 0 | 0 | 0 | 0 | 0 | 0 | - |
| **34** | 1 | 3 | 4 | 2 | 2 | 0 | 0 | 0 | 0 | 0 | 0 | 0 | - |
| **35** | 1 | 0 | 0 | 0 | 0 | 0 | 0 | 0 | 0 | 0 | 0 | 0 | - |
| **36** | 2 | 0 | 0 | 0 | 0 | 0 | 0 | 0 | 0 | 0 | 0 | 0 | - |
| **37** | 1 | 6 | 3 | 1 | 1 | 1 | 0 | 0 | 0 | 0 | 0 | 0 | - |
| **38** | 1 | 0 | 1 | 0 | 1 | 0 | 0 | 0 | 0 | 0 | 0 | 0 | - |
| **39** | 0 | 0 | 0 | 0 | 0 | 0 | 0 | 0 | 0 | 0 | 0 | 0 | - |
| **40** | 4 | 2 | 0 | 0 | 0 | 0 | 0 | 0 | 0 | 0 | 0 | 0 | - |
| **41** | 0 | 0 | 0 | 0 | 0 | 0 | 0 | 0 | 0 | 0 | 0 | 0 | - |
| **42** | 1 | 2 | 0 | 1 | 0 | 0 | 0 | 0 | 0 | 0 | 0 | 0 | - |
| **43** | 2 | 3 | 0 | 0 | 0 | 0 | 0 | 0 | 0 | 0 | 0 | 0 | - |
| **44** | 1 | 2 | 2 | 0 | 1 | 0 | 0 | 0 | 0 | 0 | 0 | 0 | - |
| **45** | 2 | 1 | 0 | 0 | 0 | 0 | 0 | 0 | 0 | 0 | 0 | 1 | - |
| **46** | 1 | 1 | 4 | 11 | 3 | 1 | 0 | 1 | 2 | 1 | 2 | 2 | - |
| **47** | 0 | 0 | 0 | 0 | 0 | 0 | 0 | 0 | 0 | 0 | 0 | 0 | - |
| **48** | 1 | 0 | 1 | 1 | 0 | 0 | 0 | 0 | 0 | 0 | 0 | 0 | - |
| **49** | 2 | 0 | 0 | 0 | 0 | 0 | 0 | 0 | 0 | 0 | 0 | 0 | - |
| **50** | 1 | 0 | 0 | 0 | 0 | 0 | 0 | 0 | 0 | 0 | 0 | 0 | - |
| **51** | 1 | 0 | 1 | 0 | 0 | 0 | 1 | 1 | 0 | 0 | 0 | 0 | - |
| **52** | 2 | 0 | 1 | 0 | 0 | 0 | 0 | 0 | 0 | 0 | 0 | 0 | - |
| **53** | 2 | 0 | 0 | 0 | 0 | 0 | 0 | 0 | 0 | 0 | 0 | 0 | - |
